# Could application of leader-member exchange theory have saved a residency mentorship program?

**DOI:** 10.1007/s40037-020-00584-2

**Published:** 2020-05-26

**Authors:** Jessica L. Bunin, Holly S. Meyer, Steven J. Durning

**Affiliations:** Department of Medicine, Uniformed Services University of the Health Sciences, Bethesda, MD USA

**Keywords:** Leadership, Mentorship, Residency program

## Abstract

Mentorship may offer protégés numerous benefits including improved self-esteem, increased interest in research, and/or enhanced productivity. Without proper planning, reflection, and evaluation, however, mentorship programs may result in undesirable consequences. In this paper we describe a mentorship program designed to improve psychosocial support and professional development for residents, that while initially successful, was terminated due to perceptions of inequity that led to strife among residents and ultimately created a toxic learning climate. Leader-member exchange theory provides a lens through which to view our program’s failure and to offer some potential solutions to mitigate such challenges for other programs. Leader-member exchange theory focuses on the importance of relationships, communication, and awareness of biases to optimize interactions between dyads such as a mentor and a protégé. We highlight opportunities during the stranger, acquaintance, and mature partnership phases that could have helped to save a residency mentorship program.

**Electronic supplementary material:**

The online version of this article (10.1007/s40037-020-00584-2) contains supplementary material, which is available to authorized users.

## The story

Mentorship programs are an important part of health professions education. They are traditionally viewed as relationships in which there is a senior individual within an organization assisting the junior person’s personal and professional development [1]. There are many benefits offered by mentorship. For example, protégés may experience improved self-esteem, increased interest in research, and/or enhanced productivity [2]. Additionally, mentors may experience increased job satisfaction, including a greater connection to their program of work. In this paper, we describe a mentorship program created to increase psychosocial support and professional development that, while initially successful, was terminated due to discord and perceptions of inequity.

While one of us was working as the Associate Program Director for an Internal Medicine Residency Program, a lack of mentorship was observed as evidenced by only three of 26 first- and second-year residents (11.5%) reporting that they had a mentor. As such, a formal mentorship program was established to improve psychosocial support and professional development for the residents. All faculty members were offered the opportunity to participate via email and announcements. Faculty members interested in mentoring provided a PowerPoint slide with training background and academic and personal interests. All residents were invited to participate and were provided a presentation including the PowerPoint slides via email and in noon conference. Residents selected their top three choices for a mentor and were matched with a faculty member. All residents were assigned one mentor and were paired with one of their top two selections. All mentors were agreeable to the pairings. Residents were encouraged to seek additional mentors if desired or if they had interests or needs that were not being addressed. The residency leadership, which included the Program Director, two Associate Program Directors, and two Chief Residents, gave the mentors an introduction to mentoring packet, which included a first meeting tool and an article about academic mentoring [3]. Residency leadership requested that the mentor-protégé pairings meet at least quarterly.

Six months after initiation of the mentorship program, residents and faculty participated in a workshop with four objectives: 1. Define mentorship, 2. Discuss traits of successful mentors and protégés, 3. Discuss obstacles to successful mentorship, 4. Compare and contrast responsibilities of mentors and protégés. The material presented at the workshop was generalized and was not based on survey results. Mentorship articles were sent to residents and faculty three times in the next six months [4–6].

**Fig. 1:**
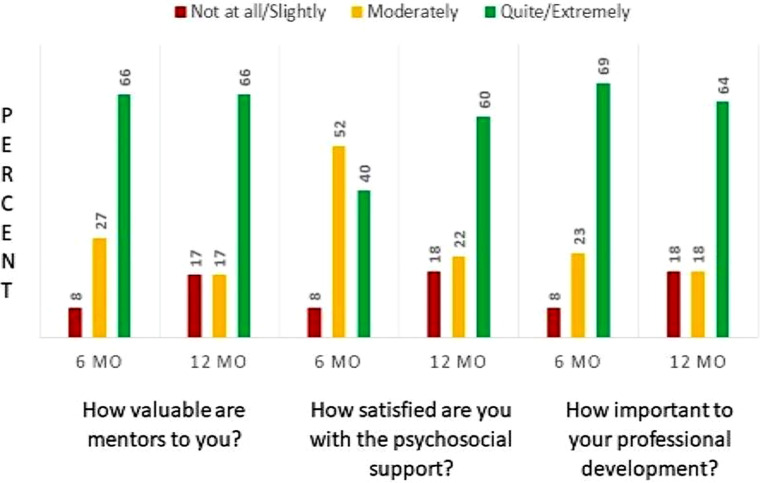
Resident perspectives on mentor relationships at 6 months (*n* = 27) and 12 months (*n* = 29) after initiation of a formal mentorship program

Resident opinions were queried via SurveyMonkey prior to the workshop and again 6 months later, a total of 12 months after the initiation of the mentorship program (See Appendix A of the Electronic Supplementary Material). The surveys were determined to be quality improvement and not research by the Institutional Review Board, there being no potential harm to respondents and as the anonymity of respondents was guaranteed. On the first survey, 39 first, second, and third-year residents were queried. Twenty-seven residents responded to the survey for a response rate of 69%; 100% of these 27 responders reported having a mentor, and 21% reported having more than one mentor. Twenty-nine residents responded to the second survey 6 months later for a response rate of 74%: 96% reported having a mentor, and 48% reported having more than one. At this time, a majority of residents felt that mentor relationships were quite or extremely valuable, they were quite or extremely satisfied with the psychosocial support received, and they felt that mentors were quite to extremely important to their professional development. Of note, a larger percentage of residents responded not at all or slightly to these questions on the second survey than the first indicating that, while a majority of residents appreciated the program, a growing percentage was dissatisfied (Fig. 1). No further formal surveys were performed after 12 months.

The residency leadership reached out to residents twice annually after the second survey via email to ask if they were happy with their mentor or if they wanted to add or to change mentors. The leadership also requested comments and ideas for improvement. Few residents reported being unhappy with their mentor, and no ideas for improvement were suggested to the residency leadership. Based on the available data, the program seemed to be successful for the first two years.

Despite the apparent success of the program, during the third year of the mentorship program, some residents filed anonymous complaints with the graduate medical education leadership at their hospital and with accrediting institutions. Complaints addressed favoritism and unfair opportunities created by the mentorship program. Residents felt that the involvement of mentors was inconsistent resulting in benefits for some, but not all, residents. For example, some faculty met with residents only in the hospital while others had study sessions at their homes or dinners for their protégés. Due to complaints, and as a means to remedy the perceived favoritism, the Program Director and the Director of Medical Education determined the best solution was to discontinue mentor meetings outside of the hospital and outside of work hours. Further, faculty were instructed to refrain from offering academic projects to specific residents, but instead to send generalized information about projects to all members of the residency. These two actions effectively led to the disintegration of the mentorship program. Other alternative solutions were not evaluated or attempted. The leadership felt that these actions would level the opportunities available to residents. This created significant conflict among the residents since the residents that were satisfied with the mentor program begrudged those who were not. A program that was designed to increase support and connection resulted in discord, animosity, and conflict.

## Surprising outcomes

The surprising outcome of our mentorship program was dissolution of the program due to the degree of dissatisfaction, tension, and resentment among a minority of residents. Additionally, we were struck by the severity of the discord that resulted from a strategic intervention designed to increase psychosocial support and professional development opportunities.

While the surveys provided evidence that a minority of residents were dissatisfied, the residency leadership did not understand the level of dissatisfaction until anonymous complaints were placed with hospital leadership and accrediting agencies. The investigation into these complaints revealed that the experiences of the protégés was indeed very diverse. Some residents met with their mentor only once or twice over the 12-month period while some residents had monthly dinners or study sessions with their mentors.

In retrospect, there were many mistakes made by the residency team. Clear guidance as to the expectations of mentors was not provided by the residency leadership. Mentors were given a general expectation of quarterly meetings with their protégé, but the specifics of the meetings were not delineated. No duration or location expectations were stated. Further, the residency leadership did not follow up individually with the mentors. The residency leadership failed to maintain formal oversight of the program and did not evaluate consistently after the first 12 months.

An additional surprising finding was that the residents felt the need to file anonymous complaints with outside agencies instead of seeking assistance from the program leadership. This was a stark reminder as to the concept of psychological size and fears experienced by residents. Despite the fact that the residents are adults, they still fear there will be retribution or consequences if they are to terminate a mentoring relationship or report dissatisfaction with a program.

## Lessons learned

A surprising lesson we learned through implementing a formal mentorship program is that an attempt at creating a supportive program can create a toxic culture for a group of learners within a residency. Therefore, even seemingly innocuous interventions should be carefully considered prior to implementation. Within our residency program, the perceptions of unfairness created a surprising level of discord among the residents, which has the potential to affect their relationships with their peers for decades to come.

Our mentorship program may have been successful if leadership had planned more appropriately and remained more engaged with the program. Leader-member exchange theory (LMX) provides one framework to prepare for, develop, reflect upon, and sustain a mentorship program. LMX focuses on the importance of relationships, communication, and awareness of biases to optimize interactions between dyads, such as a mentor and a protégé [7]. By understanding the phases of relationships as described by LMX, leaders of mentoring programs can identify areas that ought to be considered while planning and executing these programs.

LMX describes three phases of relationship development: stranger, acquaintance, and mature partnership [8]. Following the phases, we believe that creating a robust educational plan prior to and during the stranger phase, being attentive to the potential for poor matches in the acquaintance phase, and offering continued support for relationships during the mature partnership phase would have improved the overall program.

During the stranger phase, the individuals in the dyad are assessing each other and the relationship. Mentors are offering opportunities, and protégés are communicating their needs. Improved leadership communication with the mentors and protégés at this stage may ensure that expectations are consistent within the dyads. This may assist with determinations of compatibility and allow for early identification of a mismatch. An educational plan during the stranger phase could increase buy-in for the program, assist with assessment of compatibility, improve communication skills, and provide strategies for bilateral feedback and conflict management. Education regarding identification of factors indicating dissatisfaction may also prove beneficial.

During the acquaintance phase, dyads are informally negotiating the rules and boundaries of their partnership and forming mutual goals. At this time, the character of the relationship is becoming more developed, which enables clearer identification of successful and unsuccessful dyads. Applying LMX to the results of our survey during the acquaintance phase might have allowed us to identify and support struggling dyads. We unconsciously were focused on, and comforted by, the number of satisfied residents causing us to disregard the small but increasing number of dissatisfied residents. Three specific areas for improvement during this stage are increasing team building activities for dyads with potential for success, normalizing the need to terminate relationships in unsuccessful dyads, and dedicating appropriate attention and resources to on-going program evaluation and problem resolution.

For satisfied dyads that reach the mature partnership phase, as was the overwhelming majority in this program, further skills training and education could have strengthened the relationships and the program. Relationship building skills such as improving communication, conflict management, and positivity in relationships may have improved the partnerships within the program [9].

There are challenges in the development of mentorship programs that LMX does not address. It does not prescribe guidance for establishing an optimal level of structure or surveillance of a mentoring program. Additionally, it does not address how to identify individuals who are not interested in committing to mentorship or who will be dissatisfied regardless of the model created. While application of LMX suggests education, identification of dissatisfaction, increased communication, and team-building may have decreased the perceptions of favoritism, LMX does not offer a panacea for this problem.

## Moral of the story

The moral of the story is that careful consideration and planning should be undertaken prior to the development of initiatives within educational programs, even seemingly benign interventions. Considering LMX during the planning and execution of the mentorship program could have increased the likelihood of success of our program and other mentoring initiatives. The residency leadership attempted to develop a program to increase psychosocial support, provide opportunities for professional development, and ensure satisfaction for all mentors and protégés. Instead, the result was significant discord within the residency program. Improved education during the stranger phase, continued education, ongoing evaluation, and more attention to suboptimal relationships during the acquaintance phase, and continuing support through the mature partnership phase would likely have led to more mutually beneficial relationships. Further, these efforts could have avoided the repercussions of a toxic climate that were suffered by this residency. We believe that LMX theory, and more specifically, application of principles in each of these three phases, could help educators seeking to introduce or enhance mentoring programs.

## Caption Electronic Supplementary Material

Survey questions disseminated to the Internal Medicine Residents regarding the mentorship program.
