# Supplementary material for: Could application of leader-member exchange theory have saved a residency mentorship program?
Source: Perspect Med Educ. 2020 May 26;9(4):264–7. doi: 10.1007/s40037-020-00584-2 (PMC7459021; doi:10.1007/s40037-020-00584-2)
Supplement: Supplementary file 1 — Survey questions disseminated to the Internal Medicine Residents regarding the mentorship program. [file 40037_2020_584_MOESM1_ESM.docx]

**Appendix A – Mentorship Survey (administered via Surveymonkey to Internal Medicine Residents)**

1. Are you male or female?

Male

Female

2. How long have you been a physician?

1 year

2 years

3 years

3. Do you have a mentor?

Yes

No

4. Do you have a protégé?

Yes

No

5. Do you have more than 1 mentor?

Yes

No

n/a

6. Do you have more than 1 protégé?

Yes

No

n/a

7. How many times have you met with your mentor this year? (If you have more than 1 mentor please use your most prominent mentor)

None

Once

2-4 times

6-10 times

> 10 times

n/a

8. How meaningful is your mentor relationship? (If you have more than 1 mentor please use your most prominent mentor)

Not at all meaningful

Slightly meaningful

Moderately meaningful

Quite meaningful

Extremely meaningful

n/a

9. How many times have you met with your protégé? (If you have more than 1 protégé please use your most prominent mentor)

None

Once

2-4 times

5-10 times

> 10 times

n/a

10. How meaningful to you is your protégé relationship? (If you have more than protégé please use your most prominent mentor)

Not at all meaningful

Slightly meaningful

Moderately meaningful

Quite meaningful

Extremely meaningful

n/a

11. How valuable are mentor relationships to you?

Not at all valuable

Slightly valuable

Moderately valuable

Quite valuable

Extremely valuable

12. How thoroughly do you understand the value of the mentor relationship?

Not at all thoroughly

Slightly thoroughly

Moderately thoroughly

Quite thoroughly

Extremely thoroughly

13. How thoroughly do you understand a mentor's responsibilities?

Not at all thoroughly

Slightly thoroughly

Moderately thoroughly

Quite thoroughly

Extremely thoroughly

14. How thoroughly do you understand a protégé's responsibilities?

Not at all thoroughly

Slightly thoroughly

Moderately thoroughly

Quite thoroughly

Extremely thoroughly

15. How satisfied are you with the psychosocial support provided by your mentor? (If you have more than 1 mentor please use your most prominent mentor)

Not at all satisfied

Slightly satisfied

Moderately satisfied

Quite satisfied

Extremely satisfied

n/a

16. How important is your mentor relationship in your professional development? (If you have more than 1 mentor please use your most prominent mentor)

Not at all important

Slightly important

Moderately important

Quite important

Extremely important

n/a
